# Assessing DNA rates for new referrals in older adults

**DOI:** 10.1192/bjo.2021.486

**Published:** 2021-06-18

**Authors:** Aamina Cheema, Nasir Pasha, Giorgi Gurieli

**Affiliations:** 1 Little Bromwich Center, Neuropsychiatry - The Barberry Center; 2 Little Bromwich Center - Older Adults CMHT; 3 CT3 BSMHFT

## Abstract

**Aims:**

To assess DNA rates for initial assessment medical appointments offered for new referrals within an Older Adults CMHT.

To establish any correlation between waiting time and DNA rates.

To establish if the initial appointments offered were in keeping with the National guidelines (18 weeks) and our local Trust policy (1-4 weeks).

**Background:**

In the Uk 15% of adults 60 and above suffer from a mental disorder. Despite the increasing mental health burden, analysis indicate that a quarter of mental health trust received less investment from 2017 to 2018. Financial pressures have also increased appointment waiting time. The NHS has stated that by 2023 there will be a 4-week waiting time for older adult mental health services. Current national guidelines state that initial referrals should be seen within 18 weeks.

**Method:**

This is a retrospective audit looking at all first time referrals to an Older Adult CMHT in East Birmingham. 110 patients were included in this audit. Factors recorded included age, gender, reason for referral, waiting time for appointment, and whether this complies with guidelines.

Electronic patients' notes (RIO) were used for data collection.

**Result:**

Out of 110 new referrals 11 were not offered any appointments. Out of the remaining 99, 13 cancelled and 8 did not attend.

In total, 78 attended the initial appointment offered, out of which 77 were seen within 18 weeks as per national guidelines. 43 patients were seen within the 4-week period (trust policy). 1 patient was offered an appointment at 19 weeks and 3 days from the referral date. The patients who did not attend their appointments were followed up except for one, to find out the reasons of the DNA. This included 2 (physically unwell), 1 (unaware of appointment), 1 (refused), 1 (forgot), 1 (couldn't get to clinic), 1 (asthma attack). Another appointment was offered to those who could attend.

**Conclusion:**

There was no significant correlation between a longer waiting time and an increased DNA rate for first appointments. Even though the time for an initial appointment was within the NHS guidelines, only 56% of the appointments met our Trust's policy of a 4 week wait.

When discussing the results with the relevant team it was clear that a number of factors affected the waiting time including: number of available clinicians and a large catchment area.

